# EDITORIAL

**DOI:** 10.4103/0974-7788.59934

**Published:** 2010

**Authors:** Urmila Thatte

**Affiliations:** *Chief Editor, Professor and Head, Department of Clinical Pharmacology, Seth GS Medical College and KEM Hospital Mumbai 400 012, India. E-mail: urmilathatte@kem.edu*

I proudly present the first issue of the much awaited International Journal of Ayurveda Research (*IJAR*). A “felt- need” by many, the move by the Department of AYUSH, Government of India to launch this international journal has been welcomed by all.

It took a long time in coming but the time and the effort has been well worth it. Our inaugural issue, with an erudite guest editorial from Prof. MS Valiathan sets the tone for the journal. The six original articles are symbolic of the type of work we are doing in the country from a large case series of an Ayurvedic treatment “package“ for migraine to a study on the quality of traditional Ayurvedic formulation. The papers on another traditional formulation “*Ksheerbala”* and a proprietary formulation Diashis in experimental models amply indicate the possibility of bringing meticulous science to Ayurvedic medicines. There are even efforts to enhance the pharmaceutical properties of classical formulations, further illustrating the wide scope for publication in this journal. The review papers are different – one covering an important medicinal substance (backed by many references) and the other hypothetical in nature, but soundly backed by references from classical texts.

The case reports section is an area that I would greatly like to see grow. Even anecdotal reports are meaningful in practice and publication of case reports helps improve patient care – and that after all is one of the final objectives of research. With the successful launch of the National Pharmacovigilance Program for ASU drugs in India, I hope to get more case reports that will be important for students and practitioners alike.

We have introduced the section on Education as there is a need to discuss educational experiences and even research in teaching methods. The paper in this issue paves the way for policy makers to have evidence to base their decisions on.

Importantly, *IJAR* is a peer reviewed, free full-text, managed completely online Journal that is, indexed in Caspur, DOAJ, EBSCO Publishing's Electronic Databases, Expanded Academic ASAP Genamics JournalSeek, Google Scholar, Health & Wellness Research Center, Health Reference Center Academic, Hinari, SCOLOAR and Ulrich's International Periodical Directory.

I would like to specially say thank you to my editorial team, the Executive and Associate Editors, the Editorial Board members and the peer reviewers as well as the authors who have all helped make this effort possible. I look forward to more articles for forthcoming issues (we received 71 articles in 2009, of which 14 could not be accepted, many have been accepted after extensive peer review and some are in the peer review cycle.) I also look forward to a dynamic “Letter to Editor” section that should be initiated after this first issue. The encouragement from the Advisory Board, I hope, will continue always.

Till the next issue, I hope more research is translated into manuscripts.

